# Residue Adjacency Matrix Based Feature Engineering for Predicting Cysteine Reactivity in Proteins

**DOI:** 10.1016/j.csbj.2018.12.005

**Published:** 2018-12-26

**Authors:** Norman John Mapes, Christopher Rodriguez, Pradeep Chowriappa, Sumeet Dua

**Affiliations:** Program of Computer Science, College of Engineering and Science, Louisiana Tech University, 305 Wisteria St., Ruston, LA 71272, United States

**Keywords:** RAM residue adjacency matrix, Cysteine reactivity, Oxidative stress, Response pathways, Free radicals, Position specific scoring matrix, PSSM

## Abstract

Free radicals that form from reactive species of nitrogen and oxygen can react dangerously with cellular components and are involved with the pathogenesis of diabetes, cancer, Parkinson's, and heart disease. Cysteine amino acids, due to their reactive nature, are prone to oxidation by these free radicals. Determining which cysteines oxidize within proteins is crucial to our understanding of these chronic diseases. Wet lab techniques, like differential alkylation, to determine which cysteines oxidize are often expensive and time-consuming. We utilize machine learning as a fast and inexpensive approach to identifying cysteines with oxidative capabilities. We created the original features RAMmod and RAMseq for use in classification. We also incorporated well-known features such as PROPKA, SASA, PSS, and PSSM. Our algorithm requires only the protein sequence to operate; however, we do use template matching by MODELLER to acquire 3D coordinates for additional feature extraction. There was a mean improvement of RAM over N6C by 22.04% MCC. It was statistically significant with a *p*-value of 0.015. RAM provided a significant increase over PSSM with a p-value of 0.040 and an average 70.09% improvement MCC.

## Graphical Abstract

**Figure:**
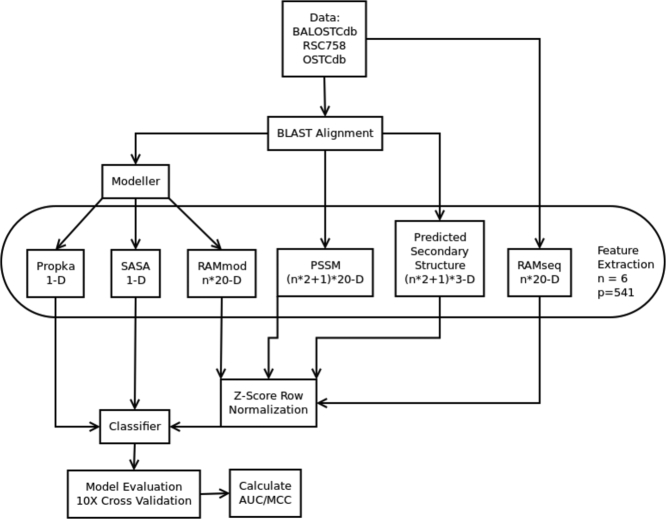

## 1 Introduction

Free radicals are known to adversely alter various biological structures (like lipids, proteins, and DNA) by introducing uneven charge distributions. If free radicals become too abundant in the body, then a condition known as oxidative stress can occur. This condition can lead to diseases such as cancer and Alzheimer's disease [38]. Oxidation susceptible cysteines in the mitochondria have been proven to play a critical role in defense against free radicals by absorbing these species [12]. Approximately 14% of the proteins studied were predicted to have mitochondrion subcellular localizations using the iLOC prediction server [35]. Cysteines also assist the body's antioxidant defense responses by inducing the glutathione response pathways [11]. Due to cysteine's critical role in combating oxidative stress, there has been growing interest in determining oxidation susceptible cysteines [13].

**Fig. 1:**
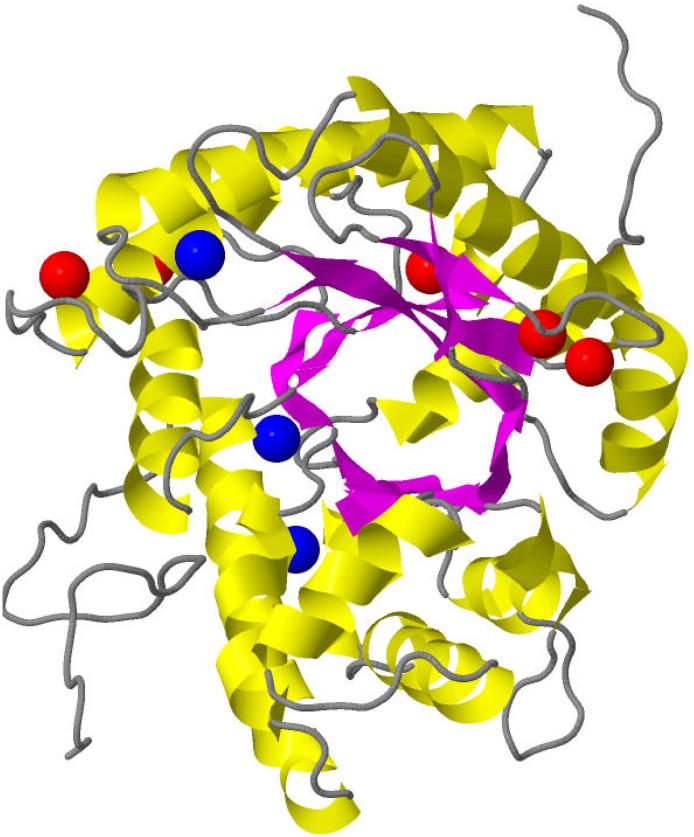
Reactive vs. Non-Reactive Sulfurs in Cysteine Residue for Protein 1ADO.

Cysteine is a unique amino acid that is a functional site in many proteins. It can be nitrosylated and glutathionylated, and can form sulfinic acid, sulfenic acid, sulfonic acid, disulfide bonds, selenocysteine, coordinate metals as well as other less common oxidations [22]. Our research and the prior works to which we compare our results focus on the former six chemistries. Some additional distinguishing properties of cysteine are its chemical plasticity, nucleophilicity, high reactivity, relative rarity, involvement in structural stabilization, catalytic activity, its status as a most common metal coordinator, and its high degree of conservation [23,26]. Cysteine plays an interesting role in redox regulation and signaling, but this role is not entirely understood. Through our prediction and scoring of cysteines that are redox susceptible, we expect that researchers can more easily understand the role of cysteine in free radical and disease states for the advancement of treatment options. Fig. 1 illustrates a typical protein that has both redox susceptible and reduced cysteines.

In this figure, the sulfur atoms of reactive cysteines in the protein 1ADO are emphasized with a blue sphere (residues 201, 338, 72 from top to bottom). The five red spheres in the protein correspond to the sulfur atoms of the non-reactive cysteines.

We hypothesize that the Residue Adjacency Matrix (RAM) based feature engineering reveals cysteine reactivity patterns for evolutionarily conserved homologous proteins. Where RAM is the n-nearest residue's distances sequentially or spatially (approximated if necessary with MODELLER) to cysteine. We also incorporate features from prior works PROPKA, SASA, PSS, and PSSM in addition to RAMseq and RAMmod. Our technique notably includes both features from a template matched 3D model (PROPKA, SASA, and RAMmod) and techniques that just require the amino acid sequence (PSS, PSSM, and RAMseq).

### 1.1 Prior Works

DISULFIND [16] and DIANNA [15] were among the first to incorporate machine learning techniques to predict the oxidation state of cysteines in proteins. They used only the amino acid sequence information as inputs. These tools first predicted which of the cysteines would form disulfide bonds via SVM classification. Their work focused solely on disulfide bonding and did not consider other oxidation states of the cysteines. After DISULFIND and DIANNA, COPA [3] was invented to classify cysteines into the four potential reactivity groups: those that form disulfide bonds, those that coordinate with metals, those that remain reduced, and those that are susceptible to reversible oxidation. Their program required 3-D coordinates, so a protein's structure must be solved using expensive techniques like X-Ray crystallography or NMR. ROCD [27], Reversibly Oxidized Cysteine Detector, was created to work similarly to COPA and also required 3-D coordinates. The goal of Lee's study was to improve the understanding of oxidative stress. [24] paper, predicted which cysteines form nitrosocysteines using solvent accessible surface area, pKa and predicted secondary structure. Hydrogen bonding and its relation to pKa was investigated for redox-sensitive cysteines to gain biochemical insights into signaling [25]. Thiol chemistry and specifically cysteine redox susceptibility were studied using quantum mechanics computational simulations for finding catalysis and regulation [28]. RSCP [7], Redox Sensitive Cysteine Prediction, was made to predict redox-sensitive cysteines. RSCP was slightly less accurate than COPA and ROCD, but the program only required the protein's amino acid sequence, eliminating the need to solve the protein's tertiary structure. Most recently, CPIPE was developed to provide a comprehensive computational platform to study various properties of cysteine residues [14]. It can work with either the sequence data alone or with both the sequence data and additional structural data. Our work is benchmarked against prior works by utilizing new features, RAMseq and RAMmod.

## 2 Materials and Methods

**Fig. 2:**
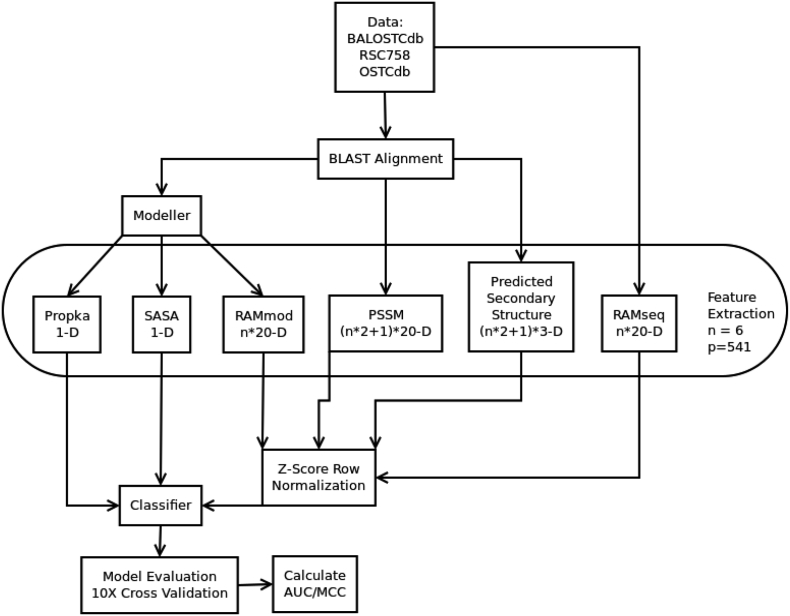
Description of the process via flowchart diagram.

Our tool takes the amino acid sequence of the protein in question as an input, compares it to databases for 3-D structural data, extracts 6 features (see Table 1) from the collected data for a total of 541 dimensions to be used in our predictors, this number may vary depending on dataset validation. In Fig. 2 first we send our data to RAMseq for feature extraction. Then we BLAST align our protein, from this alignment we calculate PSSM and PSS. Next we use the alignment for MODELLER and extract 3 sources of features PROPKA, SASA and RAMmod. For multidimensional feature sources we optionally normalize the data (required for SVM classifiers). Finally we send the extracted features to the classifier and perform 10 fold cross validation or train/test, the choice is dependent on prior works so that we can make a fair comparison. Finally we calculate metrics of success such as AUC, MCC and more. Also a confidence scores for each oxidation is made for decision making in biological workflows. Our originally engineered features, RAMseq and RAMmod, can also be applied to post-translational modification problems (PTM) generally. This is because every PTM problem has a target residue and a modification in this case it was cysteine and its modification being oxidation.

**Table 1:** Summary of features.

Feature	Original publication	Unabbreviated form	Typical dimensionality
RAMseq	This work	Residue adjacency matrix from primary sequence	≥ 120
RAMmod	This work	Residue adjacency matrix from MODELLER data	≥ 120
N6C (Sun's D)	Sun 2016	Nearest 6 cysteines distance	6
PSSM	[31]	Position specific scoring matrix	≥ 260
PSS	[33]	Predicted secondary structure	≥ 39
PROPKA	[2]	Protein pKa at cysteine sulfur	1
SASA	[4]	Solvent accessible surface area at cysteine sulfur	1

We decided to take only the amino acid sequence of the protein as input, and not require the protein's 3-D coordinates as input. Researchers have determined sequence data from around 93 million proteins whereas only 130 thousand proteins have known 3-D structures [9,10] . Our work, therefore, remains general enough to be useful to a more significant portion of the proteomics community. Although we start with only the primary amino acid sequence, we do use predictive algorithms to estimate the secondary and tertiary structures for use in some of our features e.g. predictive algorithms like MODELLER and PSSPred. If structural information of the protein exists in the RSCB Protein Data Bank, we can then integrate that information directly instead of relying on estimations from MODELLER.

### 2.1 Dataset Creation and Availability

In order to validate our methods, we decided to use three datasets: OSCTdb, BALOSCTdb, and RSC758. Sanchez and his team created the independent dataset OSCTdb (Oxidation susceptible cysteine thiol database) in 2008 by using the blastall program of the BLAST software package [6] to reduce similar records that had identities higher than 35% and e-values less than one. OSCTdb has 161 oxidation-susceptible cysteines, 301 oxidation-non-susceptible cysteines, and a total of 99 polypeptides. The BALOSCTdb (BALanced OSCTdb) dataset was created from OSCTdb by limiting the non-oxidation-susceptible cysteines to 161, which balances the number of oxidization-susceptible cysteine thiols with the number of non-oxidation-susceptible cysteine thiols. RSC758, Redox-Sensitive Cysteine 758, [7] was designed to be similar to BALOSCTdb but with a higher number of entries. RSC758 has 758 entries for both oxidized and non-oxidized cysteines. Table 2 details our dataset:

**Table 2:** Summary description of data.

Dataset	Oxidized cysteines	Reduced cysteines	Optimal RAMseq n	Optimal RAMmod n	Number of features
RSC758	758	758	12	18	901
BALOSCTdb	161	161	6	7	561
OSCTdb	161	376	5	6	521

The datasets can be found at the availability link mentioned below. The data is in the BALOSCTdb.fa, BALOSCTdb.txt, RSC758.fa and RSC758.txt, these datasets were originally from https://biocomputer.bio.cuhk.edu.hk/RSCP/download.html We are providing the CSVs with the class label, RAMseq, RAMmod, PSSM, PSS, PROPKA and SASA features for each of the three datasets as a supplemental material and online through the availability link in the file called Features 3 Datasets.zip. The feature's abbreviations in the files are the same as the for the feature importance supplementary file.

### 2.2 RAMseq

**Fig. 3:**
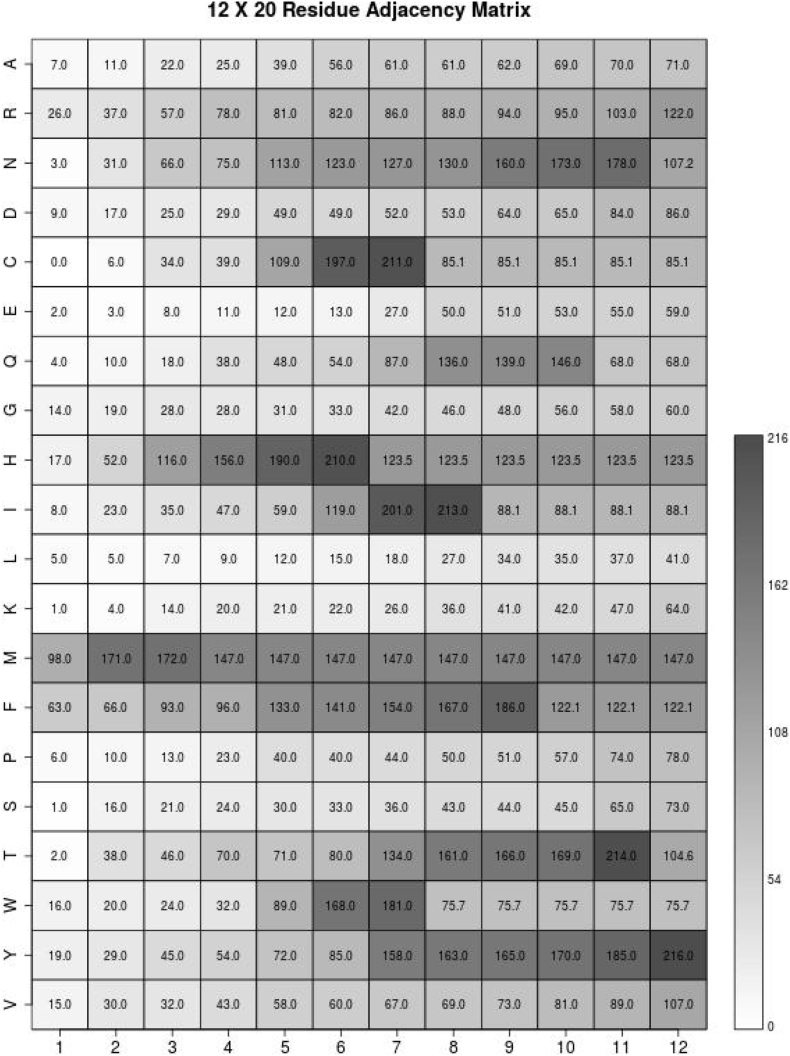
Typical protein residue adjacency matrix sequential, computed from the protein APEX_HUMAN1. Depicted is RAMseq based on cysteine 99, that is involved in reversible disulfide bonding and glutathionylation. The sequence is …ETKCSEN… where cysteine 99 is centered. Note the values do not strictly increase, because when there is not enough amino acids of the correct type the mean of the incomplete row is used for the remaining cells in the row. These matrices are used to template match each other, where similar matrices have similar redox sensitivity. Also of importance is that the matrix in this figure is transposed from the previous matrix, either is correct as long as the classifier's input is consistent across all the datapoints.

The RAMseq (Residue Adjacency Matrix from sequence data), can be calculated on the raw sequence data without any other accompanied data (like 3-D coordinates or the secondary structure). RAMseq is an n by 20 matrix formed by taking the distance of the target cysteine to the first, second, third, …, n'th closest amino acids of each type in the sequence (see Table 3 and Fig. 3 for examples). n is chosen for each dataset for optimal performance and six is a value of n that is expected to work well on an unknown dataset based on the validation experiments we performed in Table 8. RAMseq is similar to a cysteine separation profile [17] because it is a sequential index measurement, but is used for all amino acid residues instead of solely cysteine. RAMseq is a type of homology match because similar RAM matrices are correlated to similar reactivities of cysteine. The data for cysteine and tryptophan distances consistently score as one of the most prominent features. These are the two most conserved amino acids residues, as indicated by the diagonals on the BLOSUM62 matrix (a substitution matrix used for sequence alignment of proteins) [29].

**Table. 3:** Genomic residue adjacency matrix sequential, as an illustration of RAM's construction we will use the first 10 base pairs of chromosome 1 of the human genome: TAACCCTAAC. (LEFT) We are going to analyze the first cytosine. To calculate the first entry in A we subtract the interesting cystosine's position (4) from the nearest adenine's position (3) and take the absolute value: |4–3| = 1. For the second entry in A we calculate |4–2| = 2. For the third entry in A we calculate |4–8| = 4 and so on. (RIGHT) The missing nucleotides use the mean of their column, unless there are no entries in the column, in this case we use the mean of the matrix. Means provide a summary statistic in a single number that is often a used as a feature in classification problems. For example the mean of the C column is (1 + 2 + 6)/3 = 3 and G is (1 + 2 + 4 + 5 + 3 + 3 + 1 + 2 + 6)/9 = 3. It is coincidental that integer values were found for the means, any real number is a valid entry for a RAM cell.

A	T	C	G	A	T	C	G
1	3	1	N/A	1	3	1	Mean of Table = 3
2	3	2	N/A	2	3	2	Mean of Table = 3
4	N/A	6	N/A	4	Mean of Column T = 3	6	Mean of Table = 3
5	N/A	N/A	N/A	5	Mean of Column *T* = 3	Mean of Column C = 3	Mean of Table = 3

RAMseq compliments PSSM (Position Specific Scoring Matrix) in several key ways. Firstly, RAMseq measures amino acid residue proximity to the target cysteine whereas PSSM only measures the frequency of each amino acid in a specific window. Secondly, RAMseq's data can extend to positions that are further away than a PSSM reaches without oversaturating models with higher dimensionality and thereby reducing predictive capability. Thirdly, RAMseq is more compact than PSSMs: as n increases the window increases by 1, while RAMseq's noncontiguous window increases by 20*n. This means for an equivalent window size PSSM will be 20 times larger. Finally, RAMseq works directly on the inputted sequence data without relying on sequence alignments like PSSM.

The 20 × 12 matrix is an example of an optimal value of n for RSC758 its metrics of success were optimal with an n value of 12. Table 2 shows the optimal values for RAMseq as 12, 6, 5 and RAMmod as 18, 7, 6 for each of the three datasets. We used 6 as a general number that can be expected to perform well on any new dataset based on the validation results. The absolute distance in RAM is calculated by taking the index of cysteine and subtracting it from the index of the amino acid of the appropriate type and first, second, third, …, nth closest. This value is always positive whether the amino acid in question is before or after the cysteine.

(1) $${\mathrm{RAMseq}}_{i,j}=\left|C-{\mathrm{AA}}_{j}^{i}\right|$$

In Eq. (1) shown above, RAM is the value of the residue adjacency matrix, C is the index of the cysteine in question, and AA is the index of the amino acid. Furthermore, i is the row or amino acid type and j is the column or j'th nearest amino acid. For example j = 1 and *i* = 1 means the closest amino acid of the type alanine. When j = 2 and *i* = 20 it is the second nearest amino acid of the type valine and so on. When there are not enough amino acids of the specified type to fill the matrix, an ARIMA Model, Auto Regressive Integrated Moving Average, can then be utilized. ARIMA is a time series statistical technique that can provide the missing data points. Using the forecast package from R and selecting (p, d, q) according to the PACF and ACF plots gives us either a trend or a mean prediction. [34] Although means had a strong positive performance capability, trends were not found to improve scores. If two cysteines have a similar mean distance to every amino acid of a certain type, then the two likely share similar reactivity. For instance, the mean of every tryptophan's distance to cysteine was chosen as an important feature by the random forest classification model for determining reactivity. As an example, given the n shortest distances of [6,12,NA,NA,NA,NA], the final RAMseq is taken as [6,12,9,9,9,9]. In the rare case that no amino acids of a certain type are present in the protein, then the mean value of the matrix fills the row. In a review of missing value replacements we found “...Global Average Value for Numerical Attributes This method is very simple: ..numerical values are replaced with the average of all values of the corresponding attribute [37].”

### 2.3 BLAST Alignments

BLAST (Basic Local Alignment Search Tool) is a widely used and allows one to query a database for a list of similar sequences to a target sequence. Many of the features that we use (including MODELLER, PSSM and the PSS tables) require sequence alignments. All of our 3-D structure based features also implicitly rely on BLAST because MODELLER requires BLAST alignments to make its predictions on the tertiary structure of the target proteins. RAMseq was the only feature that we used which did not require a BLAST alignment to work. BLAST uses heuristic methods to search large databases for sequence matches quickly. Although it does not necessarily find the optimal alignments like the Smith-Waterman [30] or Needleman-Wunsch algorithm. BLAST works by first making a k-letter word list from the target sequence (for instance, with k = 3 and a sequence of PLDAG, BLAST would make a word list of PLD, LDA, and DAG). Next, possibly matching words are scored for each entry by use of a substitution matrix (usually BLOSUM62). Words that exceed a given threshold are designated as “high-scoring words” and are used for the remaining searches. The database is scanned for an exact match with one of the high-scoring words. On a hit, a window of the neighbors of the exact hit is expanded and scored (using the same substitution matrix from before) until the score decreases (i.e. an unlikely substitution is caught). The score of this window is recorded, and if found significant, is combined with other so-called high scoring pairs into a longer alignment. The expected score (the probability that an unrelated sequence would obtain a higher score by chance) is calculated for the alignment, and the alignments with e-values above the threshold are returned. *E*-value is the likelihood of a sequence being returned due to chance by BLAST.

### 2.4 PSSM

PSSMs (Position Specific Scoring Matrices), also known as Position Weight Matrices, are a useful data structure that captures the amino acid frequency profile of a specific window in a protein sequence. They were first introduced by Gary Stormo and his colleagues in their 1982 paper to explore patterns in E-coli nucleotide sequences. We use PSSMs as a feature in our machine learning algorithms in order to capture the amino acid compositions of sequences that are similar to our target sequence.

A PSSM is calculated from alignments at a position by dividing the observed substitutions of a specific amino acid by the expected number of substitutions. A ratio higher than one indicates that the amino acid substitution is favored. Ratios less than one indicate that that the amino acid substitution is not favored [32]. For a window size of 2*k + 1 (k positions to the left of the target cysteine, k to the right, and the target cysteine position itself) and the twenty major amino acids, we get a matrix that is twenty rows long and 2*k + 1 columns wide that can be vectorized for a total of 20*(2*k + 1) features. We chose 6 for k as found in the prior work [36]. Our PSSMs provided 260 entries for the classifier. Blastp was used from the BLAST software suite with an e-value of 0.005, and the out_pssm setting enabled.

PSSMs reveal evolutionary patterns in a local (position specific) manner. Proteins are known to generally conserve their structure as they mutate, so cysteine reactivity being conserved through small mutations is a logical extension. PSSMs can assist machine learning algorithms by adding implicit correlation between the training examples of the same class via evolutionarily homologous conserved similarities thus results from two species with a near common ancestor likely share cysteine oxidations.

### 2.5 PSS - Predicted Secondary Structure

Segments of amino acids can arrange themselves into unique local 3-D structures. These structures generally fall into three classes: alpha helices, beta sheets, or coils. In the same way that we can computationally estimate 3-D structural information from our protein sequences, we can also predict the secondary structures of the protein. We used the PSIpred software [33] to make these predictions. PSIpred use neural networks to make predictions. PSIpred requires an alignment outputted from a BLAST. We use a window size of thirteen positions (the target cysteine plus the six positions to the left and the six positions to the right) for the PSS matrix. 6 was chosen due to prior works [36]. The final matrix is then thirteen by three (the confidence score for the three classes of secondary structures) which results in a thirty-nine-dimensional feature source for the classifier once vectorized.

### 2.6 Modeller

We used the MODELLER software [18] through a Python API to estimate the 3-D structure of a protein using a technique known as comparative modeling. Comparative modeling predicts the 3-D structure of a protein based on BLAST alignments to other proteins which have a known structure. The comparative modeling algorithm consists of four general steps: fold assignment, target-template alignment, model building, and model evaluation. MODELLER first obtains an alignment of a target sequence and a database of template structures. It then automatically calculates a model containing all non‑hydrogen atoms and returns a PDB file containing the estimated 3-D coordinates of the target protein. MODELLER outputs a .pdb file that contains x, y and z coordinates for every atom in the protein. It also includes a description of each of the atoms, for instance alpha carbons through delta carbons, the name of the amino acid and the position of the amino acid. We calculated the Euclidean distance of the sulfur atom of the cysteine in question to each alpha carbon of the appropriate amino acid type up to the n closest.

In our work, we used MODELLER with the default settings. We decided to take the ten closest protein structures as our template database for MODELLER. Sometimes, the alignments that we chose to feed into MODELLER had insufficient overlap, which caused the model building to fail. To remediate this we omitted the offending template and repeated the process.

During our experiments with BALOSCTdb and OSCTdb we use the solved crystal structures because these datasets had all of them available. RSC758 had 249 cysteines with solved crystal structures and relied on MODELLER for the remaining 1267 cysteines. In order to determine the influence of crystal structures, we ran an experiment by running our classifiers using all features (including the ones derived from 3-D data like SASA, RAMmod, and PROPKA) on first the proteins with experimentally solved crystal structures and then the proteins with computationally estimated crystal structures. We measured the AUC of the ROC curve and obtained 0.732 for the 1267 MODELLER based datapoints and 0.769 for the datapoints with the 249 crystal solved cysteines. While performance dropped it is still comparable to RSCP. RSCP did not use any MODELLER based structural features. Also these results are very favorable compared to Table 4 ([7] features, a reproduction of RSCP) that also did not have MODELLER provided structural data. This indicates that structural features improve performance when they are available and should be used. MODELLER allows the classifier to make an approximation rather than an imputed value of the mean distance or a special value such as 0. An imputed value would be required for the classification when using available structural features when they are unavailable for any of the proteins studied. There is still much work to be done to predict protein folding and MODELLER is one of many attempts, it is a template based method and works reasonably well for many proteins, however we use a hybrid approach that relies on MODELLER for half of our features (SASA, PROPKA and RAMmod) and the other half that do not (RAMseq, PSSM, PSS). As solutions to the protein folding problem improve so will methods that use these predicted coordinate files such as RAMmod, PROPKA and SASA.

**Table 4:** Results obtained comparing RAM vs N6C.

Including the accompanying features SASA, pKa values, the PSSM and the PSS for both RAM and N6C.					
RAMseq + RAMmod + SASA + PROPKA + PSSM + PSS (With Most Effective Accompanying Features)					
Dataset	MCC	AUC	ACC	SN	SP
RSC758	0.383	0.743	0.687	0.573	0.800
BALOSCTdb	0.574	0.851	0.773	0.621	0.925
OSCTdb	0.422	0.763	0.703	0.547	0.859

N6C + SASA + PROPKA + PSSM + PSS (The Accompanying Features We Found to be Most Effective Paired with N6C)					
Dataset	MCC	AUC	ACC	SN	SP
RSC758	0.230	0.634	0.612	0.505	0.719
BALOSCTdb	0.539	0.828	0.754	0.590	0.919
OSCTdb	0.317	0.711	0.609	0.267	0.952

N6C + PSSM + PSS ([7] Model Features: a Reproduction of RSCP)					
Dataset	MCC	AUC	ACC	SN	SP
*RSC758*	0.272	0.669	0.627	0.450	0.805
*BALOSCTdb*	0.513	0.822	0.742	0.578	0.907
*OSCTdb*	0.345	0.733	0.683	0.646	0.721

### 2.7 RAMmod - Residue Adjacency Matrix from MODELLER Data

**Fig. 4:**
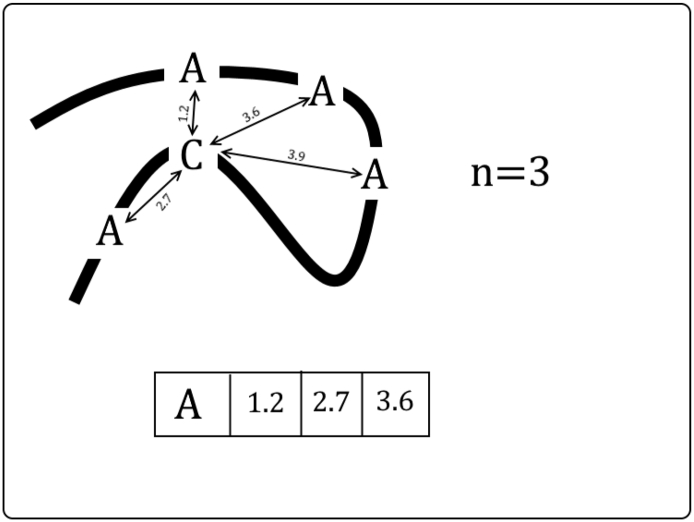
Process for obtaining the RAMmod matrix. We calculate the Euclidean distance instead of the sequential distance for all alanines to the target cysteine. The 3 smallest distances are added to the RAMmod matrix under the alanine row. This process is repeated for all the other 19 amino acids. Note the furthest sequential alanine is the nearest in euclidean distance.

RAMmod is the second original feature that we used in our work. RAMseq differs from RAMmod in the distance only. Rather than using simple positional differences like RAMseq, RAMmod uses the Euclidean distance of the target cysteine sulfur group to each of the neighboring residue's alpha carbon obtained from the protein's 3-D structure. RAMmod in Eq. (2) is the value of the residue adjacency matrix. Cx, Cy and Cz are the x, y, and z positions of the cysteine sulfur atom, and AAx, AAy, and AAz are the x, y, and z positions of the amino acid alpha carbon. i represents the ith amino acid (out of 20) and j represents the jth closest amino acid to the cysteine. Both i and j in subscripted to RAMmod are the row and column of the matrix. See Fig. 4 for an example.

(2) $${\mathrm{RAMmod}}_{i,j}=\sqrt{{\left(\mathrm{Cx}-{\mathrm{AAx}}_{j}^{i}\right)}^{2}+{\left(\mathrm{Cy}-{\mathrm{AAy}}_{j}^{i}\right)}^{2}+{\left(\mathrm{Cz}-{\mathrm{AAz}}_{j}^{i}\right)}^{2}}$$

Like RAMseq, if there are not n amino acids in the whole sequence of a specific type, then the mean of the euclidean distances for the available amino acids of the specified type is used to fill in the remainder of the row. For example, let us suppose that there are 3 tryptophan amino acids in a protein and RAMmod n is equal to 6. The euclidean distance of the analyzed cysteine to the closest tryptophan is 3.1 Å, the second closest is 5.2 and the third 7.4, then the fourth, fifth and sixth row would be (3.1 + 5.2 + 7.4) / 3 = 5.2. This assumes the residue adjacency matrix is setup where columns specify the amino acid type and rows specify the first, second, third, …, nth closest of the specified amino acid type. If no amino acids of a specific type exist, then that row is filled with the mean distance of every other amino acid type. If Modeller fails to produce a pdb, then the mean of all the crystal distances is used. Also, like RAMseq, the data for cysteine and tryptophan distances score as the most prominent features.

### 2.8 PROPKA - Protein pKa Data

We determined the pKa values of our target cysteine sulfur atoms by using the PROtein PKA software, PROPKA [1,2,8]. To determine pKa we use Eq. (3).

(3) $$\mathrm{pKa}={\mathrm{pK}}_{\text{Model}}+∆\text{pKa}$$

pKModel is set at 9.00 while??pKa was determined from hydrogen bonds, desolvation, and charge interactions. PROPKA requires 3-D coordinates, which we provide from MODELLER. The pKa values typically vary from 0.00 to 14.00, but we assign a special value of 99.99 to indicate a suspected disulfide bond. The pKa was determined to be an important feature for determining the reactivity of cysteine. It was the third-best discriminator in COPA's decision tree. A pKa value greater than nine strongly indicates the reactivity of cysteine. PROPKA determines which cysteines are suspected disulfide bonds. Because PROPKA operates on the proximity of atoms to one another we believe that when the sulfur atoms are closer than a threshold it provides the special value of 99.9. The mean without the suspected bonds was 11.2. Using 99.9, 11.2 and 0 as the value for the suspected disulfide bond our AUC's were 0.743,0.742 and 0.743. This experiment was on the RSC758 dataset.

### 2.9 SASA Data

The solvent-accessible surface area (SASA) is the surface area (measured in square angstroms) of a molecule that is available to a given solvent. We used FreeSASA [4] with the Naccess [5] settings in order to determine the SASA of our target proteins. FreeSASA requires 3-D coordinates which, again, we gather through MODELLER. The SASA value of a protein is helpful for determining the reactivity of a target cysteine. Proteins with similar SASA scores are likely to have similar redox sensitivity. SASA was the second most crucial discriminator in COPA's decision trees. Values >1.3 Å squared tend to indicate a reactive cysteine.

### 2.10 Normalizing the Data

Before providing the features to the machine learning algorithms, we experimented with applying both *Z*-score normalization (Eq. (4)) and min-max normalization (Eq. (5)) to our data. We normalized on the sets of each feature array at each row. Features with a dimensionality of one (like SASA) were not normalized. Z-score normalization was found to be more effective than min-max normalization. Normalizing the entire feature matrix or rather the entire row or the entire column was ultimately found to be less effective. Standard column normalization was required for the SVM classifier to function properly. Random forest did not need any normalization. In Eqs. (4), (5), Z_i_ is a normalized feature of a data point, feature_i_ refers to a specific feature of a data point and feature_sd_, feature_mean_, feature_min_ and feature_max_ being the standard deviation, mean, min and max respectively of the feature type for that datapoint. PSSM or RAM is an example of a feature type. Feature types are normalized independently of other features for each data point.

(4) $$Z_{i}=\frac{{\mathrm{feature}}_{i}-{\mathrm{feature}}_{\mathrm{mean}}}{{\mathrm{feature}}_{\mathrm{sd}}}$$

(5) $$Z_{i}=\frac{{\mathrm{feature}}_{i}-{\mathrm{feature}}_{\min}}{{\mathrm{feature}}_{\max}-{\mathrm{feature}}_{\min}}$$

### 2.11 Classification and Metrics of Performance

We experimented with classification using a random forest algorithm, an SVM, and KNN. Random forest was ultimately found to be the most effective. We validated this using 10 fold cross validation with ROC curves in Fig. 10 of Section 3.5. Random forests are resistant to overfitting due to bootstrapping and a limit on the number of features considered at each split. Pruning the trees (by setting the max_depth parameter) in the random forest can help to prevent overfitting. Random forests can also rank features by their importance. A collection of binary decision trees each evaluate the reactivity of our target cysteine. The average of the trees is then evaluated to provide a confidence score. The confidence scores are used to create a receiver operating characteristic curve, ROC. This curve plots the sensitivity against the false positive rate (1 - specificity). The area under this curve, AUC, is a single number that describes the ability of the classifier to separate the data into two classes (in our case, cysteines that undergo oxidation and those that do not). A confusion matrix is then made to determine the Matthew's Correlation Coefficient using Eq. (6). The source code for this calculation is found in the availability link under RSC758.py or any of the other dataset's py files. For a calculation of maximum MCC threshold, an iteration through the threshold values is done until the highest MCC is found.

(6) $$\mathrm{MCC}=\frac{\text{TP}\times \text{TN}-\text{FP}\times \text{FN}}{\sqrt{\left(\mathrm{TP}+\mathrm{FP}\right)\left(\mathrm{TP}+\mathrm{FN}\right)\left(\mathrm{TN}+\mathrm{FP}\right)\left(\mathrm{TN}+\mathrm{FN}\right)}}$$

## 3 Results and Discussion

We now present the evidence which show the ability of RAMseq and RAMmod to determine the oxidation susceptibility of cysteines more accurately than previous methods. In the sections below, we use RAM to refer to the combined feature matrix of RAMseq and RAMmod, which are the original features of this work.

### 3.1 RAM Vs. N6C

**Fig. 5:**
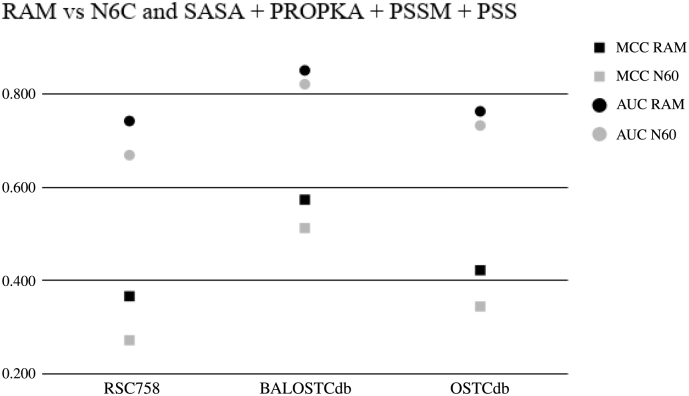
Matthew's Correlation Coefficient and Area Under the Receiver Operating Characteristic Curve Comparison for RAM vs N6C each with their accompanying features. The reason for the full feature set is because a fair comparison of normal usage can be made.

The feature N6C is like RAMseq except *n* = 6 and only cysteines are considered. N6C provided the highest discriminative ability of redox susceptible cysteines before this work. Fig. 5 shows the MCC and AUC of the two sources of features. We replicated the work of [7] and made a comparison to RAM. There was a 22% mean improvement for all three datasets over [7] Model Features compared to RAM with a *p*-value of 0.015. Furthermore, there was a 27% improvement over N6C with both having the accompanying features we used compared to our results, the p-value was not significant at the alpha level of 0.05 but was 0.055. See Fig. 5 and Table 4 for the detailed results.

### 3.2 RAM Vs. PSSM

**Fig. 6:**
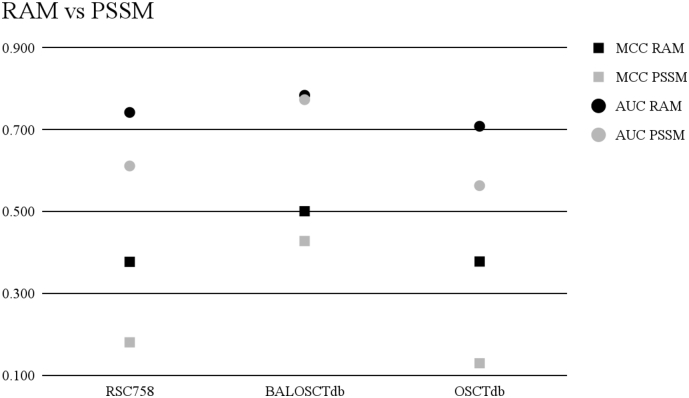
Matthew's Correlation Coefficient and area under the receiver operating characteristic curve. No other features are included.

PSSM is a frequently used method in proteomics and genetics. Because RAM has been shown below to outperform PSSM, there is a great deal of promise for using RAM in broader applications. We provide the radar chart in Fig. 8 for comparing the comparison of RAMseq to PSSM. PSSM performed higher on only one dataset (BALOSCTdb) while RAMseq performed better on the other two datasets (RSC758 and OSCTdb). See Fig. 6 and Table 5 for the detailed results.

**Table 5:** Results obtained comparing RAM vs PSSM.

The comparison of PSSM to RAM without any other features					
RAMseq + RAMmod					
Dataset	MCC	AUC	ACC	SN	SP
RSC758	0.378	0.743	0.676	0.496	0.856
BALOSCTdb	0.501	0.785	0.748	0.683	0.814
OSCTdb	0.378	0.709	0.674	0.472	0.875

PSSM					
Dataset	*MCC*	*AUC*	*ACC*	*SN*	*SP*
RSC758	0.181	0.612	0.586	0.745	0.426
BALOSCTdb	0.428	0.774	0.711	0.627	0.795
OSCTdb	0.130	0.564	0.567	0.752	0.383

PSSM is a broadly used feature set that has been in existence for >30 years. RAM alone was 70% higher in terms of MCC compared to PSSM alone. This difference was significant with a *p*-value of 0.040.

### 3.3 Prior Works

**Fig. 7:**
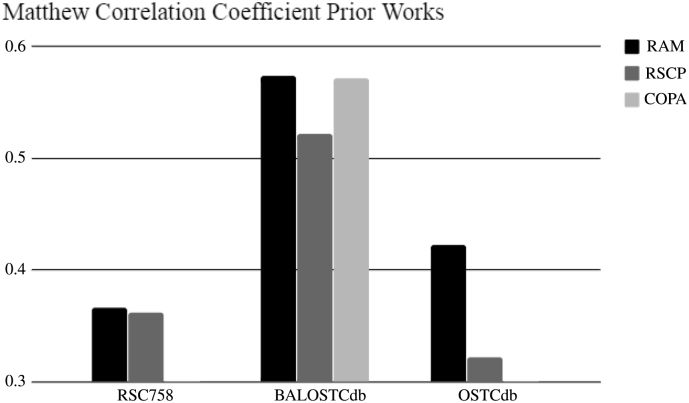
A comparison of RAM with all supplementary features against the other two methods (RSCP and COPA) on our 3 datasets (RSC758, BALOSTCdb, and OSTCdb). RAM has the highest MCC of all methods on all datasets.

In the following section, we make comparisons between RAM, RSCP and COPA. RSCP's primary contribution was the ability to use sequential features without the need of solved 3-D structural data. RSCP, therefore, is more broadly applicable than algorithms like COPA, which requires a PDB to predict cysteine redox susceptibility. However, COPA's accuracy was higher than RSCP's accuracy. RAM is a hybrid approach that accepts structural features but can use MODELLER predictions when only sequential data is provided. See Fig. 7 and Table 6 for the detailed results of the experiments.

**Table 6:** Comparison of RAM to prior works COPA and RSCP.

Comparing RAM to prior works					
RAM					
Dataset	MCC	AUC	ACC	SN	SP
RSC758	0.383	0.743	0.687	0.573	0.800
BALOSCTdb	0.574	0.851	0.773	0.621	0.925
OSCTdb	0.422	0.763	0.703	0.547	0.859

RSCP					
Dataset	MCC	AUC	ACC	SN	SP
RSC758	0.362	0.727	0.679	0.602	0.756
BALOSCTdb	0.522	0.821	0.761	0.770	0.752
OSCTdb	0.322	NA	0.629	0.789	0.561

COPA					
Dataset	MCC	AUC	ACC	SN	SP
RSC758	NA	NA	NA	NA	NA
BALOSCTdb	0.572	0.823	0.786	0.776	0.795
OSCTdb	NA	NA	NA	NA	NA

RAM had higher MCC and AUC than RSCP and COPA. The average MCC improvement across the three datasets for a RAM vs RSCP comparison was 14% with a *p*-value of 0.064. This is not significant at the alpha level of 0.05 but it suggests that RAM is a better source of features than prior works. Because COPA has performance metrics for only one dataset, no statistical analysis is possible, but RAM outperforms on MCC and AUC metrics of success.

### 3.4 Prior Features Vs. RAM

**Fig. 8:**
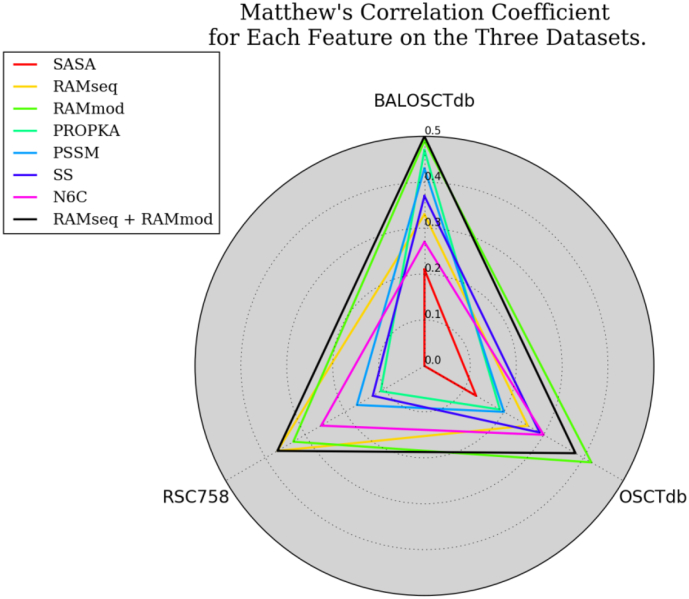
Note that the radar chart shows that RAM sits on the outer edges of the chart compared to other features. This indicates that the features have higher performance on every dataset compared to all features in prior works. The results in this figure are for each feature used alone.

**Fig. 9:**
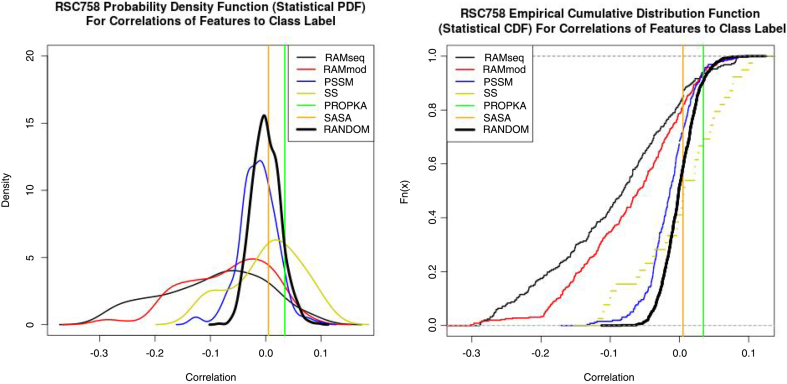
(LEFT) Shown above is the probability density function approximated using the statistical software R. The density function in the stats package was used with default parameters. Note the vertical lines for SASA and PROPKA these are one-dimensional features; therefore the correlation pdf is a vertical line. The probability of a correlation existing in a range is found by taking the integral between the min and max of any two points. With this in mind, the plot indicates that RAMmod and RAMseq have a significant probability of a correlation with the class label, albeit negative. This suggests that the goodness of the features can be observed using non-classification tools such as ordinary least squares (OLS). It is important to note that each curve has an area below it equal to one. (RIGHT) By transforming the PDF to a CDF using R we can see the probability of a feature's correlation being equal or less than a particular value. We make this transform so that we can run a statistical test, the Two Sample Kolmogorov-Smirnov test or simply KS test. Our *p*-value is <2.2e-16 comparing RANDOM to RAMseq and RANDOM to RAMmod.

RAMseq and RAMmod are distinguishable like all prior features. In Fig. 8 RAMseq + RAMmod sit on the outside for the dataset BALOSCTdb indicating their combined performance is optimal to either alone. However for RSC758 the features RAMseq and RAMmod taken together is equivalent to RAMseq taken alone. Finally for OSCTdb, RAMmod outperforms the two taken together. It is possible to use one or both of the features for classification. See Fig. 8, Fig. 9 for the results.

### 3.5 Choosing an Optimal N for RAMseq and the Results of Using 6,6 for RSC758

A comparison between the different values of n was performed and results reported in Table 8. The n of 6 was found to be effective in prior research [36] It is a coincidence that RAMseq and RAMmod were fairly close to the number 6 for PSS and PSSM as seen in Table 2. We used 6 as a general number that can be expected to perform well on any new dataset based on the results. Table 7 shows the effect of using an n of 6 for both RAMseq and RAMmod.

**Table 7:** Results from adjusting the *n* parameter on both RAMseq and RAMmod.

RAMseq + RAMmod + SASA + PROPKA + PSSM + PSS					
Dataset and n	MCC	AUC	ACC	SN	SP
*RSC758 6,6*	0.314	0.711	0.646	0.460	0.831
*RSC758 12,18*	0.367	0.743	0.679	0.569	0.789

**Table 8:** AUC by Varying RAMseq n for an Optimal RAMmod n.

AUC as a function of RAMseq n with RAMmod n fixed at its optimal value												
N	*4*	*5*	*6*	*7*	*8*	*9*	*10*	*11*	*12*	*13*	*14*	*15*
RSC758												
AUC	0.729	0.737	0.736	0.737	0.734	0.740	0.739	0.744	0.743	0.740	0.741	0.741

BALOSCTdb												
AUC	0.854	0.856	0.851	0.851	0.849	0.855	0.841	0.839	0.844	0.845	0.842	0.839

OSCTdb												
AUC	0.760	0.763	0.729	0.717	0.701	0.693	0.680	0.686	0.673	0.669	0.675	0.673

We defaulted to a value of n = 6 but found RAMseq *n* = 12 and RAMmod *n* = 18 gave us the highest metrics of success for the RSC758 dataset. Optimizing the values of n for RAMseq and RAMmod increased the AUC by 4.5% and MCC by 16.8%. However, optimizing the value of n may lead to overfitting the data.

We performed ten fold cross validation and obtained n values that were optimal for RAMseq and RAMmod, reported is the AUC for each RAMseq n on the three datasets. Table 2 shows the optimal values for RAMseq as 12, 6, 5 and RAMmod as 18, 7, 6 for each of the three datasets across all the metrics of success.

### 3.6 Choosing an Optimal Matthew's Correlation Coefficient and Classifier

**Fig. 10:**
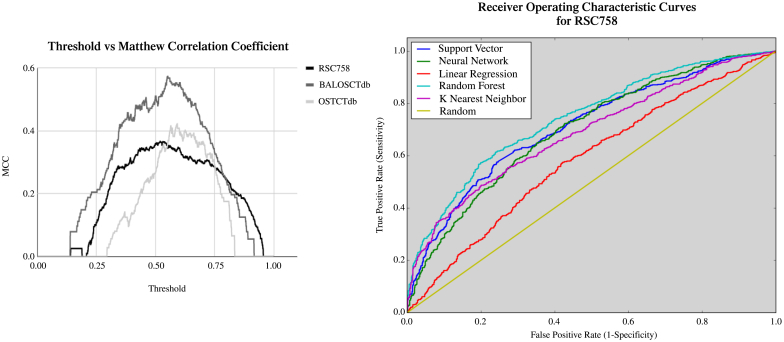
Matthew's Correlation Coefficient as a function of threshold and ROC Curve for Classifiers. (LEFT) We chose the optimal threshold for MCC after varying classifier, classifier parameters and feature parameters for optimal AUC. (RIGHT) By analyzing five different classification schemes, including OLS and a random baseline we can visualize the differences in sensitivity and specificity of each of these algorithms. Notably, random forest is the best performer as shown by its higher and shifted leftward appearance. A perfect curve would follow the left and top side.

Adjusting the classification threshold of the confidence scores (the value at which we decide to classify a data point as the other label) for our classifiers ensures the optimal performance of our final tool. In this section, we show how varying the classification threshold affects the model performance. Fig. 10 (LEFT) plots the MCC as a function of a threshold. The optimal value occurs at the peak of the curve. Fig. 10 (RIGHT) shows the receiver operating characteristic curve for 5 different classifiers on RSC758.

A comparison between the classifiers RF, SVM, KNN and more was done in Fig. 10. The ROC curve for random forest was the furthest left and topmost. This indicates that for the binary classification problem any confidence threshold separating the classes provides a better metric of success (sensitivity and specificity). As the classifier threshold is decreased (to the right and top of the curve), the classifier becomes more sensitive (labeling oxidizing cysteines as oxidizing) while sacrificing specificity (labeling non-oxidizing cysteines as non-oxidizing) and vice versa.

## 4 Conclusion

Our results clearly show the benefit of applying our original features, RAMseq and RAMmod, to machine learning approaches for cysteine reactivity predictions. By every metric on which we scored, a feature system including RAM outperformed a system using N6C. N6C was the best feature to date. Our work achieved state-of-the-art accuracies, yet only required the primary amino acid sequence of the target proteins and integrated structural features when available.

With regards to model evaluation and reproducibility of the results. The model evaluation is illustrated in Fig. 2. It is standard ten fold cross validation, with the exception of OSCTdb that was a trained with RSC758 and evaluated with OSCTdb (this is the same method in RSCP and we can make a fair comparison by training and testing likewise). The reproducibility of the calculations of MCC are in Eq. (6) and was calculated using the python package sklearn, AUC was calculated similarly. The source code for the calculations can be found under the availability link. The results have been reproduced on a separate Windows computer by installing Anaconda, using Git to download the data and files and running the RSC758.py, BALOSCTdb.py and OSCTdb.py in a Spyder IDE (part of Anaconda). For the AUC and MCC calculations all that is needed for sklearn is the binary class label (oxidized/reduced or 1/0) and the classifier predicted probability that varies between 0 and 1. Linux and Macintosh follows an identical workflow.

RAM is readily comparable to a PSSM. Because of the prevalence of PSSM's in current literature, a similar feature such as RAM could be useful in improving the accuracies of a great deal of proteomic and genetic machine learning techniques. PSSM does well conducting local searches but will frequently fail on distant conserved regions due to its small window size. RAM can handle these distant conserved regions quite well. RAM is also useful for a broad class of problems, notably post-translational modifications because there are residues of interest that need to be classified and neighboring residues in these problems. RAM can be modified to work with DNA. For DNA, the matrix is 4*n, and has the rows A, T, C and G. Future work where RAM data is used for DNA may complement PSSM for genetic problems as has been shown in this work for cysteine reactivity.

Biologically either of these two new sources of features measure homology of proteins. An interesting experiment showed us that the most conserved amino acids were the best features of RAM (see supplementary material). In other words how far the most conserved amino acids such as tryptophans and cysteines (these two amino acids are most conserved based on BLOSUM62 substitution matrix diagonal) are from the cysteine in question allowed us to choose the most homologous cysteines. For example, if a mouse protein retains several tryptophans from its common ancestor with humans then the oxidation state of a cysteine at a similar distances is likely to be shared. For instance the protein insulin in mice and humans shares many of the same cysteine oxidations at the 3 disulfide bridges (Cysteines 31–96, 43–109 and 95–100 Uniprot P01308 and P01326) and several of the amino acids' distances to those cysteines are shared, thus RAM would identify this conservation and therefore the oxidized cysteines. Of interest is that experiments relevant to mice can be mapped to humans by using RAM or from one species to another when they have conserved amino acids from a common ancestor.

## Availability

Data and Replicable Results are provided at https://github.com/johnmapesjr/RAM
